# Parents experiences of discharge readiness from a Swedish neonatal intensive care unit

**DOI:** 10.1002/nop2.71

**Published:** 2016-11-09

**Authors:** Christina Larsson, Ulrika Wågström, Erik Normann, Ylva Thernström Blomqvist

**Affiliations:** ^1^ Neonatal Intensive Care UnitUniversity HospitalUppsalaSweden; ^2^ Department of Women's and Children's HealthUppsala UniversityUppsalaSweden

**Keywords:** discharge, neonatal intensive care unit, parents’ experience, qualitative content analysis, questionnaire study, transition to home

## Abstract

**Aim:**

The aim of this study was to describe how parents experienced the support at, and preparation for discharge from, the NICU and how they experienced the first time at home.

**Design:**

A qualitative design with quantitative elements was applied.

**Methods:**

A questionnaire study. Data were analysed using qualitative content analysis with quantitative elements.

**Results:**

The majority of included parents felt adequately prepared for going home and sufficiently supported during the first period home. Negative experiences were related to lack of time for preparation, lack of support and information, especially about the infant's food intake, breastfeeding, and tube feeding, and lack of follow‐up counselling post discharge. This study supports that parents who are closely involved in their infant's care at the NICU, and who stay with the infant at the NICU around the clock, are well prepared for the transition to home.

## 1 Background

Parents of infants requiring care at a neonatal intensive care unit (NICU) can have difficulty in participating in their infant's care because of the infant's complex care needs and the NICU environment. Monitoring equipment and care giving activities around the infant can form a barrier that complicates the attainment of the parental role (Raines & Brustad, 2012). The separation between parents and their infant, due to the NICU environment and routines, makes it difficult for the parents to feel and act as parents (Fenwick, Barclay, & Schmied, 2008). This separation leads the parents through a particular transition to parenthood, which is different from what parents with a healthy, full term infant experience (Shin & White‐Traut, 2007).

The transition from the NICU to home can be a turbulent experience for parents and can cause stress and anxiety. Parents experience more difficulty in recognizing changes in the infant's condition and performing the infant's medical care on the first day at home compared to when they were staying at the NICU (Raines & Brustad, 2012). Parents of infants who have received care at the NICU are often insufficiently prepared for their infant's discharge and have unanswered questions prior to NICU discharge (Sneath, 2009). This contributes to increased anxiety within the family and an increased need for health and medical care as visits and phone calls (Smith, Hwang, Dukhovny, Young, & Pursley, 2013). Parents who are allowed to be actively involved in, and take over, their infant's care at the NICU feel empowered and strengthened in their parental role, and have a sense of control over the situation (Heinemann, Hellstrom‐Westas, & Hedberg Nyqvist, 2013). Many care giving tasks, such as feeding and holding, allow parents to interact with, and get to know, their infant (Duhn, 2010; Goulet, Bell, St‐Cyr, Paul, & Lang, 1998). Parents are also helped to bond with their infant and feel like parents if the NICU staff continuously provide information about the infant's health status, how the NICU and its equipment work, and if there are any changes in the infant's care.

It is important that NICU staff effectively plan discharge together with the parents to support the parents when they return to home after hospital stay (Rowe & Jones, 2010). Few studies examine parents’ experiences and perceptions of discharge from the NICU. Healthcare professionals need to understand parental experiences so that they are able to meet parents’ needs and provide appropriate support to increase their caring confidence after discharge.

### 1.1 Aim

The aim of this study was to describe how parents of infants receiving care at an NICU experienced the preparation and support at the NICU before their infant's discharge, and their first few days at home.

## 2 Method

### 2.1 Design

A qualitative design with quantitative elements was applied.

### 2.2 Ethical considerations

This study was part of an ongoing quality improvement project at the NICU involved. Permission to perform the study was obtained from the NICU's medical director. According to Swedish law, no ethical approval is required for this kind of study. The authors followed the ethical principles outlined by the Helsinki declaration (World Medical Association Declaration of Helsinki 2013). The letter inviting parents to participate in the study contained information about the voluntary nature of participation and how to contact the researchers and their supervisor if any questions arose. To ensure total anonymity, the questionnaires were without identification and could not be linked to specific participants.

### 2.3 Study setting

This study was conducted at a 21‐bed NICU at Uppsala University Hospital, Uppsala, Sweden. The NICU consists of three intensive care rooms with four beds each. Next to every infant's bed, there is an adult bed for one parent (Heinemann et al., 2013), which allows continuous parental presence with the infant. There are also nine family rooms where, as soon as the infant is no longer critically ill or in need of ventilator support, both the parents and the siblings can stay with the infant to carry out his or her care day and night. All parents at the NICU are encouraged to stay with their infant and participate in the infant's care as much as the situation allows.

At the NICU concerned, discharge planning began when an infant was admitted to the NICU and actively involved the parents in their infant's care (Nyqvist & Engvall, 2009). Through early Kangaroo Mother Care (Blomqvist, Ewald, Gradin, Nyqvist, & Rubertsson, 2013), separation between the infant and his or her parents was reduced as far as possible. There were no specific guidelines, based on weight or age, for when an infant could leave the NICU, but it was desirable that the infant should be older than a postmenstrual age of 34 weeks at discharge with a satisfactory weight gain. It was also important that the parents wanted to leave and felt comfortable with leaving the NICU. All parents of infants born before 35 gestational weeks or infants who had been critically ill participated in a special parental education programme approximately one week before leaving the NICU. This parental education programme consisted of information given by a registered nurse regarding sudden infant death syndrome, about how to secure the infant in a car seat, how to avoid infections, and also information about, and practical training in, cardiopulmonary resuscitation (CPR) for infants. Parents were given both written and verbal information and the information session took about an hour. As part of the programme, the infant performed a “breathing registration,” which consisted of monitoring by electrocardiography (ECG) and saturation of blood oxygen during one night. A neonatologist determined if the infant had mature breathing and no apneas.

### 2.4 Participants

Infants (*n* = 93) were identified through the medical records. Parents of infants cared for at the NICU were asked to participate in the study. The period of data collection was February and March 2013. Inclusion criteria were that the infant should have been cared for at the NICU for at least 1 week during 2012, was a singleton, and was discharged directly home from the NICU. Parents whose infant died after the NICU stay or who needed an interpreter during hospitalization were excluded.

### 2.5 Data collection

To fulfil the aim of the study, a questionnaire was developed by the authors based on their clinical experience and current research. The questionnaire consisted of 11 questions: six concerned background information about the infant, two comprised both closed and open‐ended response options, and three were free‐text questions. At the end of the questionnaire there was possibility to attach a separate sheet of paper if the parents required more space to answer the questions. A letter stating the purpose of the study and inviting the parents to participate in the study was sent to the infants’ home address together with the questionnaire and a pre‐paid response envelope. To ensure the parents’ and infants’ anonymity, the questionnaires were not coded. A reminder letter was sent to all participants 4 weeks after the first mail.

Sixty‐six (71%) questionnaires were completed. Thirty‐two (48%) were answered by the mother, four (6%) by the father, and 27 (41%) by both parents together. In three questionnaires, parents did not state who had filled in the questionnaire. Not all parents answered all questions in the questionnaire, but according to parents’ answers, 26 girls and 37 boys were born between gestational weeks 25 and 44; altogether 39 were born prematurely, and 39 had no siblings. Two of the infants were cared for only in an intensive care room at the NICU, 26 were cared for only in a family room together with the parents, and 36 infants were cared for in both types of care settings. The infants’ total length of stay at the NICU was between 7–86 days.

### 2.6 Data analysis

The parents’ responses to the open‐ended and free‐text questions were analysed using qualitative content analysis, based on the description by Graneheim and Lundman (2004). The method included the following steps: The responses were written down verbatim in a Word document and carefully read through several times to obtain an understanding of the content. The text was divided into meaning units, which means that constellations of words containing one idea or one piece of information were identified. No shortening of the text was done because the answers were already brief. Meaning units were labelled with codes based on the content. Codes with similar content were grouped together to summarize the data. Groups of codes that shared common content were identified into categories and subcategories. The analysis procedure was mainly performed by the two first authors, (C.L and U.W) who independently identified meaning units and then compared findings. A few differences in interpretation were discussed until consensus was reached. All authors discussed the preliminary results and consensus was reached through common reflection and discussion.

## 3 Results

When asked if the parents felt adequately prepared for discharge home, 55 (83%) responded “yes,” three (5%) responded “no,” and eleven (17%) answered the question in free text. When asked if they felt they received sufficient support from the NICU for the first few days at home, 49 (74%) responded “yes,” no parent answered “no,” and 18 (27%) parents answered in free text.

Three categories and eight subcategories were identified in the analysis of parents’ experience of discharge from the NICU. An overview of categories and subcategories is presented in Table 1.

**Table 1:** Overview of categories and subcategories

Categories	Subcategories
The experience of preparing for discharge	Ambivalent feelings
	Factors positively affecting discharge preparation
	Factors negatively affecting discharge preparation
The experience of the first period at home	Ambivalent feelings
	Facilitating factors
	Difficulties and negative feelings
Suggestions for improving the facilitation of the initial period at home	Suggestions for improving the NICU discharge preparation
	Requests for follow‐up after discharge

NICU, neonatal intensive care unit.

### 3.1 The experience of preparing for discharge

#### 3.1.1 Ambivalent feelings

Many parents felt ambivalent about their preparation to go home and leave the NICU. The majority felt ready to go home, but experienced some anxiety particularly at the first permission of discharge. The ambivalence parent′s experienced was related to feelings of safety, of not being practically prepared, and not being fully mentally prepared. They stated that they felt both ready and not. The first permission of discharge was sometimes associated with much anxiety. Monitor equipment and nursing staff at the NICU had given feelings of safety and when the infant was about to be without this extra supervision it felt strange and worrisome. However, on the whole they felt safe to go home and knew they could manage to take care of their infant on their own:

Practically, we were well prepared. But not mentally. (Participant 24)

#### 3.1.2 Factors positively affecting discharge preparation

Several parents emphasized the importance of being told their infant had been declared healthy. This created a sense of security before going home with the infant and they trusted the NICU staff's statement that their infant was healthy.

Several parents mentioned that the medical examinations conducted on their infant created security for going home. The parental education and medical information provided was appreciated. The NICU staff's guidance and support was considered positive by the majority of parents. Many considered the staff to be knowledgeable and the knowledge members of staff shared with the parents provided them with self‐confidence in the care of their infants. Positive encouragement from the staff made parents feel confident. Several parents stated that staff were calm and provided guidance customized to their needs.

Many parents highlighted the opportunity of participating in their infant's care and felt that staying with the infant at the NICU helped them feel prepared for going home. Their participation and responsibility was gradually increased during the NICU stay, which was experienced as positive. Parents appreciated the staff's attitude to the infant's care and that staff considered the parents as the infant's primary caregiver:

That we should take care of our son was natural to them, and that strengthened us. (Participant 33)

Several parents said that the gradual transition to home through day and/or night permission enhanced the feeling of being well prepared.

#### 3.1.3 Factors negatively affecting discharge preparation

Some parents did not feel fully prepared for discharge, mainly because they did not have time to make the necessary practical preparations at home. Other parents lacked emotional support from the nursing staff. They stated that the discharge felt unplanned and unstructured from the NICU staff's side. Some parents also experienced a lack of support at the NICU, particularly regarding tube feeding and breastfeeding.

### 3.2 The experience of the first period at home

#### 3.2.1 Ambivalent feelings

Many parents had ambivalent feelings during the initial period at home. Although it felt good to be home, they also felt some concerns. The first period at home was a major adjustment, especially considering the difference from the NICU environment (with lack of monitoring devices for the infant) and that they now had full responsibility for their infant's care:

Of course we were somewhat extra concerned for him in the initial period at home, although it was still great to be home. (Participant 8)

Many parents thought it was great to go home, away from the NICU environment. They realized they could take care of their infant by themselves, and at home they could create their own routines calmly.

#### 3.2.2 Facilitating factors

Several parents reported that the time they had been together with their infant at the NICU facilitated their first period with the infant at home. Factors that facilitated the first time at home were support and help from the NICU staff and the opportunity to be involved in the infant's care during the NICU stay:

I was at the NICU all the time, so at home it worked at least as well. (Participant 5)

Many parents appreciated the support available from the NICU during the first period at home, especially being able to call the NICU. It also felt good to revisit the NICU, where they had the opportunity to ask questions.

#### 3.2.3 Difficulties and negative feelings

Most parents felt some kind of concern during the first period at home. A common source of concern was the unfamiliarity of being without the monitoring equipment and NICU staff. Several parents were particularly worried about their infant's breathing. Moreover, the infant's food intake and weight gain worried many parents, as they were concerned about whether the infant got enough food. The source of the concern was sometimes practical because some infants were still tube fed and some parents were afraid they would do something wrong related to this:

The first few days at home were a little bit scary without the NICU staff near as we were used to. We checked frequently that he was breathing! (Participant 19)

Several parents stated it was hard to find sufficient time at home for all that needed to be done. The practicalities of infant feeding took time, and it was hard to find time for themselves, such as time to eat and sleep. Some parents felt isolated at home. At first, they avoided people and felt vulnerable. There was a fear that their infant would suffer from an infection if exposed to too many other people.

### 3.3 Suggestions for improving the facilitation of the initial period at home

Several parents stated that they were satisfied with the NICU care and had no suggestions for improving the initial period at home. Many said that a person cannot be completely prepared for the new situation at home, after having an infant admitted to the NICU:

I don't think you can really be sufficiently prepared; it's something so totally new in your life, so you will never be prepared enough. (Participant 1)

#### 3.3.1 Suggestions for improving the discharge preparation

Several parents asked for more preparation before discharge. They wanted a planning meeting prior to discharge in which they, together with the staff, could go through what had been prepared at home and what else they needed to do. Some parents felt that discharge was too early; they thought it would have been better if they had been able to go outside the NICU with their infant before being discharged. According to some, a checklist would facilitate discharge preparation.

Some parents suggested that a gradual reduction in healthcare intervention would have facilitated the mental preparation for going home.

Many parents suggested that more information and support from the NICU would have facilitated their first time at home. Most requested additional information relating to breastfeeding and the infant's food intake.

#### 3.3.2 Requests for follow‐up after discharge

Several parents wanted a follow‐up after discharge, including follow‐up care in terms of more, or longer, return visits; home visits; and supportive conversations after discharge. Some parents felt that support calls could have helped them find their parental roles at home more quickly.

## 4 Discussion

In this study, a majority of parents felt they were prepared for going home with their infant. This result differed from previous studies (Smith et al., 2013; Sneath, 2009) reporting that parents did not feel prepared for going home from the NICU. One reason for this difference could be that some studies were performed in countries with different conditions to Sweden in terms of parental leave. Also, the NICU in this study had routines that benefited parents’ preparation for discharge. For example, the opportunity for parents to be with their infants during the NICU stay and to be involved in the infant's care appeared to promote parents’ preparation for discharge. Baylis et al. (2014) described that parents’ involvement in their infants’ care appeared to depend on the NICU environment and routines, such as parents’ opportunity to stay with their infant around the clock during the whole NICU stay or NICU guidelines dictating when parents were allowed to start providing care. Most NICU families consider themselves prepared for discharge, especially when they feel confident about the neonatologist's decision, their infant's health, and their home environment (Smith, Young, Pursley, McCormick, & Zupancic, 2009).

Sneath (2009) report that parents often have unanswered questions before going home from the NICU. In addition, Smith, Dukhovny, Zupancic, Gates, and Pursley (2012) report that in their study, NICU families feeling prepared for discharge often had an infant with a higher discharge weight and had a discharge nurse who was known to them. Unprepared families report that their neonatologist did not examine their infant before discharge and have more problems with the infant's feeding and formulas (Smith et al., 2012). Although the majority of parents in our study reported that they felt adequately prepared for going home, some appeared to have still unanswered questions at discharge. Several parents requested more information regarding their infant's food intake, breastfeeding and tube feeding.

The transition from the NICU to home is often a turbulent experience with feelings of stress and anxiety (Raines & Brustad, 2012). The parents in this study had ambivalent feelings before discharge and during the first period at home. They had many positive feelings but some worries of various kinds at the same time.

In order for parents to feel confident and have the skills to adequately take care of their infant after discharge, they need to be involved in their infant's care throughout the NICU stay (Griffin & Abraham, 2006). This was supported in this study, as the majority of parents mentioned that participation in their infant's care, and support and supervision from the NICU staff were contributing factors to their sense of preparation, which facilitated their first period home.

Several parents requested enhancements for facilitating the first few days at home, including more information while at the NICU, and more counselling and follow‐up from the NICU after discharge. Similarly, in a study by Smith et al. (2012), parents shared their ideas about how the transition home could be improved: they requested a discharge nurse known to them; clear discharge criteria and processes; and that the family should be informed as soon as possible if there were any changes to the discharge plan.

If parents of infants at an NICU are insufficiently prepared for their infant's discharge, this can contribute to increased anxiety for them and an increased need for health care visits (Smith et al., 2013). Staff at the NICU can facilitate parents’ transition from the NICU to home through avoiding the separation of an infant in need of NICU care and its parents, by actively involving the parents in their infant's care at an early stage.

### 4.1 Limitations

In this questionnaire study, data were analysed using qualitative content analysis to provide a broad overview of the results. During the whole analytical process, the authors discussed and reflected upon the preliminary results until consensus was reached, which strengthens the trustworthiness of the results (Graneheim & Lundman, 2004). Relevant quotations are included in the results section to allow the reader to judge the authenticity and credibility of the analysis. The response rate was 71%. The questionnaire was concise, which could have contributed to the favourable response rate. Another possible reason for the favourable response rate may be that many parents considered it important to answer the questionnaire so as to influence the transition from the NICU to home for other parents. It may be advantageous to use both closed and open‐ended answer alternatives in questionnaires: closed response options are often easier for the respondent to answer (Jakobsson & Westergren, 2005); however, in open‐ended responses, the respondent has the opportunity to provide a balanced and ample answer.

One factor that could be considered as both strength and weakness of the study was that the included infants varied in gestational age at birth and length of hospital stay. Some infants needed high‐tech NICU care; others did not. However, parents of all infants cared for in a NICU have one factor in common: they have a difficult first time with their infant in an environment with unfamiliar medical equipment and NICU staff involvement. Therefore, the strength of the study was that the result covered parents’ different experiences within this broad sample group. One weakness of the study was the difficulty in determining which experiences related to specific health needs of the infant. Unlike Smith et al. (2012), no analyses regarding demographics or diseases were performed in this study. Another weakness was the use of questionnaires; it would have been considerably better to use interviews. Interviews provide the possibility for follow‐up questions which could have improved the understanding of the responses received. Another limitation was the small sample size, collected from a single NICU, making the results difficult to generalize. However, this was not the aim of the study as this study was part of an ongoing quality assurance activity indicating that parents who are closely involved in their infant's care during the infant's whole NICU stay, and who stay with the infant at the NICU around the clock, are well prepared for the transition to home.

## 5 Conclusion

The majority of parents felt prepared for leaving the NICU with their infant. Factors favouring the parents’ sense of being prepared for going home and affecting their experience of the first period at home were being present during, and involved in, the infant's care at the NICU; the infant's being medically examined and declared healthy; the parents getting sufficient medical and practical information; and the transition to home occurring gradually. At discharge, several parents wished they had had more information about breastfeeding, tube feeding, and the infants’ food intake; they also would have liked emotional support and follow‐up counselling after discharge. This study supports that if parents of infants admitted to the NICU are closely involved in their infant's care and allowed to stay with the infant at the NICU around the clock, they will be well prepared for the transition home.

## Conflict of interest

We have no conflict of interest to declare.

## Author contributions

CL, UW, EN and YTB were all responsible for the study conception and design; CL, UW and YTB performed the data collection; CL, UW, EN and YTB were all were participated in the data analysis and for the drafting and performing critical revisions to the paper for important intellectual content; YTB supervised the study.

## References

[nop271-bib-0001] Baylis, R. , Ewald, U. , Gradin, M. , Nyqvist, K. H. , Rubertsson, C. , & Blomqvist, Y. T. (2014). First‐time events between parents and preterm infants are affected by the designs and routines of neonatal intensive care units. Acta Paediatrica, 103, 1045–1052. 24923236 10.1111/apa.12719

[nop271-bib-0002] Blomqvist, Y. T. , Ewald, U. , Gradin, M. , Nyqvist, K. H. , & Rubertsson, C. (2013). Initiation and extent of skin‐to‐skin care at two Swedish neonatal intensive care units. Acta Paediatrica, 102, 22–28. 23072448 10.1111/apa.12056

[nop271-bib-0003] Duhn, L. (2010). The importance of touch in the development of attachment. Advances in Neonatal Care, 10, 294–300. 21102171 10.1097/ANC.0b013e3181fd2263

[nop271-bib-0004] Fenwick, J. , Barclay, L. , & Schmied, V. (2008). Craving closeness: A grounded theory analysis of women's experiences of mothering in the Special Care Nursery. Women Birth, 21, 71–85. 18463016 10.1016/j.wombi.2008.03.006

[nop271-bib-0005] Goulet, C. , Bell, L. , St‐Cyr, D. , Paul, D. , & Lang, A. (1998). A concept analysis of parent‐infant attachment. Journal of Advanced Nursing, 28, 1071–1081. 9840879 10.1046/j.1365-2648.1998.00815.x

[nop271-bib-0006] Graneheim, U. H. , & Lundman, B. (2004). Qualitative content analysis in nursing research: Concepts, procedures and measures to achieve trustworthiness. Nurse Education Today, 24, 105–112. 14769454 10.1016/j.nedt.2003.10.001

[nop271-bib-0007] Griffin, T. , & Abraham, M. (2006). Transition to home from the newborn intensive care unit: Applying the principles of family‐centered care to the discharge process. The Journal of Perinatal & Neonatal Nursing, 20, 243–249. 16915057 10.1097/00005237-200607000-00012

[nop271-bib-0008] Heinemann, A. B. , Hellstrom‐Westas, L. , & Hedberg Nyqvist, K. (2013). Factors affecting parents’ presence with their extremely preterm infants in a neonatal intensive care room. Acta Paediatrica, 102, 695–702. 23590800 10.1111/apa.12267

[nop271-bib-0009] Jakobsson, U. , & Westergren, A. (2005). Enkätmetodik – en svår konst (Construction of questionnaires – a difficult task). Vård i Norden, 25, 72–73.

[nop271-bib-0010] Nyqvist, K. H. , & Engvall, G. (2009). Parents as their infant's primary caregivers in a neonatal intensive care unit. Journal of Pediatric Nursing, 24, 153–163. 19268237 10.1016/j.pedn.2008.07.006

[nop271-bib-0011] Raines, D. A. , & Brustad, J. (2012). Parent's confidence as a caregiver. Advances in Neonatal Care, 12, 183–188. 22668692 10.1097/ANC.0b013e318256efd5

[nop271-bib-0012] Rowe, J. , & Jones L. (2010). Discharge and beyond. A longitudinal study comparing stress and coping in parents of preterm infants. Journal of Neonatal Nursing, 16, 258–266.

[nop271-bib-0013] Shin, H. , & White‐Traut, R. (2007). The conceptual structure of transition to motherhood in the neonatal intensive care unit. Journal of Advanced Nursing, 58, 90–98. 17394620 10.1111/j.1365-2648.2006.04194.x

[nop271-bib-0014] Smith, V. C. , Dukhovny, D. , Zupancic, J. A. , Gates, H. B. , & Pursley, D. M. (2012). Neonatal intensive care unit discharge preparedness: Primary care implications. Clinical Pediatrics, 51, 454–461. 22278175 10.1177/0009922811433036

[nop271-bib-0015] Smith, V. C. , Hwang, S. S. , Dukhovny, D. , Young, S. , & Pursley, D. M. (2013). Neonatal intensive care unit discharge preparation, family readiness and infant outcomes: Connecting the dots. Journal of Perinatology, 33, 415–421. 23492936 10.1038/jp.2013.23

[nop271-bib-0016] Smith, V. C. , Young, S. , Pursley, D. M. , McCormick, M. C. , & Zupancic, J. A. (2009). Are families prepared for discharge from the NICU? Journal of Perinatology, 29, 623–629. 19461593 10.1038/jp.2009.58

[nop271-bib-0017] Sneath, N. (2009). Discharge teaching in the NICU: Are parents prepared? An integrative review of parents’ perceptions. Neonatal Network, 28, 237–246. 19592365 10.1891/0730-0832.28.4.237

[nop271-bib-0018] World Medical Association Declaration of Helsinki (2013). World Medical Association Declaration of Helsinki: Ethical principles for medical research involving human subjects. Journal of the American Medical Association, 310, 2191–2194. 24141714 10.1001/jama.2013.281053

